# Quality of Life, Fear of COVID-19, Psychological Distress, and Resilience Among Individuals with Chronic Conditions: Evidence from the Later Phases and Aftermath of the COVID-19 Crisis

**DOI:** 10.3390/diseases14040134

**Published:** 2026-04-08

**Authors:** Elpida Stratou, Georgia-Nektaria Porfyri, Stavros Antonopoulos, Afroditi Biziou, Aikaterini Kalogeropoulou, Katerina Theodorou, Kalliopi Kalogeropoulou, Aikaterini Kyriaki Timotheou, Maria Kapouralou, Aikaterini Gamvroula, Maria Saridi

**Affiliations:** 1 Department of Psychiatry, General Hospital of Argolida, 21231 Argos, Greece; geoporfyri@hotmail.fr (G.-N.P.); up1118636@upatras.gr (A.B.); katia-kal@hotmail.com (A.K.); katerina_mateika@hotmail.com (K.T.); kalliopikalogeropoulou97@gmail.com (K.K.); kt231498@students.euc.ac.cy (A.K.T.); kapourm2@gmail.com (M.K.); gamvroulak@gmail.com (A.G.); 2 Department of Occupational Therapy, University of West Attica, 12243 Aigaleo, Greece; 3 Emergency Department, General Hospital “H Agia Sofia” Children’s Hospital, 11527 Athens, Greece; paidiatros.antonopoulos@gmail.com; 4 Department of Educational Sciences and Social Work, University of Patras, University Campus, 26504 Rio, Greece; 5 Department of Psychology, School of Humanities, Social and Educational Sciences, European University Cyprus, 2404 Nicosia, Cyprus; 6 Department of Nursing, School of Health Sciences, University of Thessaly, 41500 Larissa, Greece; 7 Health Administration, Hellenic Open University, 26335 Patras, Greece

**Keywords:** chronic conditions, quality of life, psychological distress, depression, resilience, COVID-19, mental health

## Abstract

Background/Objectives: The COVID-19 pandemic posed significant challenges to quality of life, particularly for individuals living with chronic physical and/or mental conditions. Psychological factors such as fear of COVID-19, psychological distress, and resilience may be associated with quality-of-life outcomes during prolonged public health crises. This study aimed to examine quality of life and its psychological correlates among individuals with chronic conditions during the later phases and aftermath of the COVID-19 pandemic crisis. Methods: A cross-sectional study was conducted among 293 adults with chronic physical and/or mental conditions attending the General Hospital of Argolida, Greece. Participants completed validated self-report measures assessing quality of life (MVQOLI), fear of COVID-19 (FCV-19S), depression, anxiety, and stress (DASS-21), and psychological resilience (CD-RISC-25). Descriptive statistics, Spearman correlation analyses, and multivariable regression models were used to examine associations and identify factors associated with quality-of-life domains. Results: Higher levels of fear of COVID-19 and depressive symptoms were significantly associated with poorer quality of life across multiple domains. Depressive symptoms showed consistent negative associations with functional, interpersonal, transcendent, and overall quality-of-life scores. In contrast, psychological resilience was positively associated with interpersonal, transcendent, and overall quality of life. Regression analyses showed that depressive symptoms were negatively associated with overall quality of life, while resilience was independently associated with better quality-of-life outcomes. Conclusions: Psychological distress, particularly depressive symptoms and fear related to COVID-19, was associated with lower quality of life among individuals with chronic conditions during the later phases and aftermath of the COVID-19 crisis. Psychological resilience was positively associated with better quality-of-life outcomes, underscoring its relevance for supporting well-being during and after public health crises.

## 1. Introduction

The COVID-19 pandemic has had a major impact on global health, affecting both physical and mental well-being across populations [1]. Individuals with pre-existing chronic conditions faced particular difficulties during this period, including increased health concerns related to the virus and disruptions in their usual medical care and follow-up [2]. Moreover, public health restrictions, social isolation, and ongoing uncertainty related to the pandemic were associated with increased levels of psychological distress, with higher rates of depression, anxiety, and stress reported worldwide [3,4,5,6,7].

The negative effects of the COVID-19 pandemic on quality of life have been widely reported, especially among people living with chronic health conditions [8,9,10,11]. Quality of life is a broad concept that reflects not only physical health, but also emotional well-being, daily functioning, interpersonal relationships, and personal meaning in life [9,11]. During prolonged periods of stress, such as a global pandemic, these aspects of life may be affected alongside mental health symptoms [8,10].

An increasing body of research emphasizes the role of psychological resilience, defined as the ability to adapt and maintain psychological balance in the face of stressful experiences [12]. Resilience develops through a combination of individual characteristics, life experiences, social support, and environmental influences, and involves biological, psychological, emotional, social, and spiritual dimensions [13,14]. Higher levels of resilience are associated with better mental health outcomes, more effective coping with chronic illness, and better overall quality of life [14,15,16]. Examining resilience together with psychological distress and fear related to COVID-19 may therefore provide a more complete understanding of how individuals with chronic conditions continued to experience infection-related burden and psychological strain across the later phases and aftermath of the COVID-19 crisis, and how different psychological factors relate to their quality of life [17,18].

The present cross-sectional study examined the relationships between quality of life, psychological distress, fear of COVID-19, and resilience in a sample of 293 individuals with chronic conditions. The study also examined the factors associated with various domains of quality of life, with a particular focus on the role of resilience in relation to psychological distress during the later phases and aftermath of the COVID-19 crisis. Based on the existing literature, we hypothesized that higher levels of fear of COVID-19 and psychological distress would be associated with poorer quality-of-life outcomes across multiple domains, whereas higher levels of psychological resilience would be associated with better quality of life, potentially attenuating the negative associations between psychological distress and quality-of-life domains across this transitional period.

## 2. Materials and Methods

### 2.1. Study Design

This was a cross-sectional observational study including 293 patients receiving follow-up care at the General Hospital of Argolida, Greece. An a priori sample size calculation was not performed because recruitment was based on a convenience sampling approach during the study period. To evaluate the statistical adequacy of the sample, a post hoc power analysis was performed. With a final sample size of *N* = 293 and a significance level set at α = 0.05, the study achieved a statistical power (1 − β) of 0.98 for detecting medium effect sizes. This analysis suggested that the available sample was adequate to detect statistically significant associations within the study sample; however, it should not be considered a substitute for an a priori sample size calculation. The study protocol was approved by the Institutional Review Board of the General Hospital of Argolida (approval number [15-20/04/2021]; approval date: 20 April 2021). Printed questionnaires were distributed to a sample of patients during routine follow-up visits, accompanied by a brief explanation of the study objectives. Participation was voluntary and anonymous, and all participants provided written informed consent before completing the questionnaires. The estimated time for completion of the printed questionnaire was approximately 20–25 min. Participants were encouraged to take breaks if needed, and research staff were available to assist when necessary. Completed questionnaires were collected in the same manner as they were distributed. The study was conducted between September 2021 and September 2023, spanning the later phases of the COVID-19 pandemic and the transition to its aftermath. The inclusion criteria were: (i) willingness to participate, (ii) a documented diagnosis of a chronic physical and/or mental condition, and (iii) active follow-up at the General Hospital of Argolida.

### 2.2. Measures

#### 2.2.1. Socio-Demographic Questionnaire

Participants provided anonymous information on their socio-demographic and health characteristics, including age, gender, marital status, number of children, household size, educational level, employment status, type of work during COVID-19 restrictions, area of residence, chronic physical or mental health conditions, medication adherence, and routine medical follow-up. Lifestyle factors were also recorded, such as smoking, alcohol consumption, physical activity, sedentary behavior and daily use of computers and mobile devices. Additionally, participants reported sources of social support, COVID-19 infection and vaccination status, trust in the healthcare system and medical staff regarding pandemic management, use of protective measures against COVID-19, and expectations regarding the pandemic’s resolution.

#### 2.2.2. Fear of COVID-19 Scale (FCV-19S)

Participants completed the 7-item self-administered Fear of COVID-19 Scale (FCV-19S) to assess fear related to COVID-19 [19]. Each item is rated on a five-point Likert scale (1 = strongly disagree to 5 = strongly agree), with total scores ranging from 7 to 35. Higher scores indicate greater fear of COVID-19. No specific cutoff score was applied in the present study. The Greek version of the FCV-19S was translated and validated by Tsipropoulou et al. [20], demonstrating good internal reliability.

#### 2.2.3. Depression Anxiety Stress Scale-21 (DASS-21)

Participants completed the Depression Anxiety Stress Scale-21 (DASS-21), a 21-item self-report instrument derived from the original 42-item DASS [21]. The scale assesses three related negative emotional states: depression, anxiety and stress, with 7 items per subscale. Items are rated on a four-point Likert scale (0 = did not apply to me at all to 3 = applied to me very much or most of the time), with subscale scores ranging from 0 to 21, and a total score ranging from 0 to 63. Higher scores indicate greater severity of symptoms. Established severity cutoffs for the depression, anxiety, and stress subscales were used for descriptive interpretation. The DASS-21 has demonstrated good convergent and discriminant validity and a reliable factor structure across clinical and nonclinical populations [21,22]. The Greek version was validated by Lyrakos et al. [23], showing good internal reliability.

#### 2.2.4. Connor–Davidson Resilience Scale (CD-RISC-25)

Participants also completed the Connor–Davidson Resilience Scale (CD-RISC-25), a 25-item self-administered questionnaire designed to assess psychological resilience, initially developed by Connor and Davidson [24]. Each item is rated on a five-point Likert-type scale (0 = not true at all, 4 = true nearly all the time), with total scores ranging from 0 to 100. Higher scores indicate greater resilience. The scale includes five subscales: personal competence/tenacity, trust in one’s instincts/tolerance of negative affect, positive acceptance of change/secure relationships, control, and spiritual influences. An example item is: “I am able to adapt when changes occur.” No specific cutoff score was applied in the present study. The Greek version was validated by Dimitriadou and Stalikas [25], demonstrating high internal consistency.

#### 2.2.5. Missoula–Vitas Quality-of-Life Index (MVQOLI)

Finally, participants completed the Missoula–Vitas Quality-of-Life Index (MVQOLI), a 25-item self-administered questionnaire evaluating quality of life [26]. The scale assesses five dimensions: symptoms, functional status, interpersonal relationships, emotional well-being, and transcendence. Each dimension includes five items capturing: (1) perceived status, (2) satisfaction with the status, and (3) the importance of the dimension to the overall quality of life. Items are rated on a five-point Likert scale, with higher scores indicating better quality of life. The five MVQOLI domains range from −30 to +30, while the transformed total score ranges from 0 to 30. No validated cutoff values were applied in the present study. The Greek version was validated by Theofilou et al. [27], demonstrating high internal consistency.

#### 2.2.6. Statistical Analysis

Quantitative variables were expressed as mean (standard deviation [SD]) and median (interquartile range [IQR]), while categorical and ordinal variables were presented as absolute and relative frequencies. The Kolmogorov–Smirnov test was employed to assess the normality of the data distribution. To explore associations between continuous variables, Spearman’s correlation coefficients (rho) were utilized. Stepwise multiple linear regression analysis was performed to identify factors associated with the total MVQOLI scale and its individual dimensions. The regression models included participants’ demographic and clinical characteristics, fear of COVID-19, resilience, and anxiety, depression, and stress (as measured by the DASS-21 scale). Due to the non-normal distribution of the dependent variables, a logarithmic transformation was applied prior to analysis. Adjusted regression coefficients (β) and their corresponding standard errors (SE) were reported. Data were screened for completeness and consistency prior to analysis. No data imputation was performed. Records with missing values were excluded from the relevant analyses. All tests were two-tailed, with statistical significance set at *p* < 0.05. Statistical analyses were conducted using IBM SPSS Statistics (version 27.0).

## 3. Results

### 3.1. Sample Characteristics

The socio-demographic and clinical characteristics of the study population (*N* = 293) are presented in Table 1. The sample was predominantly female (55.6%) and consisted primarily of younger and middle-aged adults, with 38.7% aged 18–40 years and 37.0% aged 41–59 years. This age distribution may reflect the inclusion of both chronic physical and chronic mental health conditions, some of which commonly require long-term follow-up beginning in early or middle adulthood.

Most participants were married or cohabiting (56.5%), had at least one child (58.9%), and lived in households of three or more members (52.2%). Most also resided in urban environments (67.8%). In terms of education and employment, 34.5% had completed secondary education, 30.4% had completed primary or lower secondary schooling, 26.4% were unemployed, and 22.2% were retired. Among those working during the COVID-19 period, 52.8% continued to work on-site.

Regarding clinical characteristics, 62.5% reported a chronic physical condition and 29.0% reported a long-standing mental health disorder. Adherence to medical treatment was high (87.1%), and 82.4% reported regular follow-up attendance. More than half of the participants (52.5%) received medical monitoring through private healthcare facilities.

In terms of lifestyle and support, 53.0% reported a sedentary lifestyle, 40.1% were current smokers, and 41.2% reported mobile device use exceeding 3 h daily. Spouses were identified as the primary source of support by 38.9% of participants, followed by friends (25.3%) and children (20.1%). The use of mobile phones and computers for more than 3 h per day was significantly associated with age (*p* < 0.001). Participants aged 18–40 years exhibited the highest rates of use (46.8% for computers and 61.6% for mobile phones), compared with those aged 41–59 years (34.6% for both devices), and particularly compared with participants aged 60 years and older (9.9% and 18.3%, respectively).

Regarding COVID-19-related characteristics, 6.9% had a history of infection, and 51.4% had been vaccinated. Trust in healthcare professionals was generally higher than trust in the healthcare system overall. Nearly half of the participants (45.5%) were unsure whether the pandemic would be managed promptly, while 26.0% believed that the virus would be controlled soon.

### 3.2. Levels of Quality of Life, Fear of COVID-19, Psychological Distress, and Resilience

Descriptive statistics for quality of life, fear of COVID-19, psychological distress, and resilience are presented in Table 2. The mean total MVQOLI score was 17.8 ± 2.8. Domain mean scores were 7.4 ± 8.4 for symptoms, 3.3 ± 10.0 for functioning, 9.2 ± 13.1 for interpersonal relationships, −2.4 ± 11.8 for well-being, and 10.1 ± 11.9 for transcendence. The mean Fear of COVID-19 score was 16.8 ± 6.0. Mean DASS-21 subscale scores were 4.8 ± 5.5 for depression, 4.2 ± 4.9 for anxiety, and 6.1 ± 5.2 for stress, while the mean total DASS-21 score was 15.0 ± 14.8. The mean resilience score was 68.5 ± 16.8.

### 3.3. Correlations Between Quality of Life, Fear of COVID-19, Psychological Distress, and Resilience

Correlation analyses using Spearman correlation coefficients are presented in Table 3. The functional domain was negatively associated with fear of COVID-19, depression, and total DASS-21 score. Interpersonal quality of life showed positive correlations with resilience and negative correlations with fear of COVID-19, depression, anxiety, and stress. The well-being domain was negatively associated with fear of COVID-19, while the transcendent domain was positively related to resilience and negatively associated with fear of COVID-19, depression, and anxiety. The total quality-of-life score was positively associated with resilience and negatively correlated with fear of COVID-19 and depression. Overall, greater resilience and lower psychological distress were associated with higher quality of life.

### 3.4. Predictors of Quality-of-Life Domains

Regression analyses examining factors associated with quality-of-life domains are presented in Table 4. Symptom-related quality of life was higher among participants living in a village, island, or other non-urban areas compared with those in small or large cities. Lower functional quality of life was associated with greater fear of COVID-19 and higher levels of depression. Depression was also negatively associated with interpersonal quality of life, whereas higher resilience was positively associated with better interpersonal functioning. Well-being was negatively associated with greater fear of COVID-19. Transcendent quality of life was negatively associated with depression and the presence of chronic physical illness, while resilience was positively associated with this domain. Finally, total quality of life was positively associated with lower depression scores and higher resilience.

## 4. Discussion

The present study investigated quality of life and its psychological correlates among patients with chronic physical and/or mental conditions during the later phases and aftermath of the COVID-19 crisis. The findings indicate that fear of COVID-19 and depressive symptoms were significantly associated with poorer quality of life across several domains, while psychological resilience was positively associated with more favorable quality-of-life outcomes. Specifically, higher levels of depression and fear were linked to poorer functional, emotional, and overall quality of life, whereas greater resilience was associated with better interpersonal, transcendent, and total quality-of-life scores. These results highlight the complex interplay between psychological distress, adaptive capacities, and quality of life during a prolonged public health crisis.

Fear of COVID-19 was negatively associated with multiple dimensions of quality of life, particularly functional status, emotional well-being, and overall quality of life. This finding is consistent with previous studies showing that elevated COVID-19-related fear among individuals with chronic conditions is associated with poorer physical and psychological quality-of-life outcomes [28,29]. Heightened fear has been shown to be associated with greater psychological distress, largely through increased anxiety, depression, stress, and intolerance of uncertainty, which may undermine daily functioning and subjective well-being [30]. Moreover, prolonged exposure to pandemic-related uncertainty and health threats has been linked to increased psychological burden at the population level, with particularly adverse effects among vulnerable groups, and may also be associated with poorer overall quality of life [4,7,31,32]. However, given the cross-sectional design of the present study, these associations should not be interpreted as indicating directionality, as poorer quality of life may also contribute to greater fear and psychological burden.

Depression was consistently associated with poorer functional, interpersonal, emotional, and transcendent quality-of-life domains, as well as with lower overall quality of life. This finding is consistent with extensive pre-pandemic evidence showing that depression is consistently associated with reduced quality of life in both chronic physical illness and mental health populations [33,34]. During the COVID-19 pandemic, depressive symptoms appear to have intensified, particularly among individuals with chronic diseases, as highlighted by systematic reviews and meta-analyses reporting substantially higher prevalence rates compared with the general population [35,36]. Pandemic-related factors, including fear of contagion, reduced self-care behaviors, social isolation, and disruptions in access to healthcare services, may have further contributed to the association between depression and poorer daily functioning and subjective well-being [37,38,39].

Psychological resilience was positively associated with interpersonal, transcendent, and overall quality of life, highlighting its positive association with adaptation to stressors across the later phases and aftermath of the COVID-19 crisis. Higher resilience has been consistently linked to better emotional adjustment, adaptive coping strategies, and improved quality of life in individuals facing chronic illness or traumatic events [40,41]. Recent studies during the pandemic indicate that resilience may mitigate the adverse associations of fear, depression, and stress, and may be linked to sustained well-being and functional capacity. Among individuals with chronic diseases, resilience was associated with better disease management, reduced psychological distress, and enhanced social interactions [10]. Furthermore, research in general populations confirms that individuals with higher resilience and well-being experienced lower levels of distress and perceived threat from COVID-19, even when accounting for demographic and socioeconomic factors [42].

From a clinical and public health perspective, the present findings highlight the importance of addressing psychological distress, particularly depressive symptoms and fear related to COVID-19, in the care of individuals with chronic conditions. Interventions aimed at enhancing psychological resilience and supporting adaptive coping strategies may help improve quality-of-life outcomes during periods of prolonged uncertainty and health-related stress. Nevertheless, the cross-sectional design of the study limits causal interpretations, and the direction of the observed associations cannot be determined, while the reliance on self-report measures may introduce reporting bias. In addition, because data collection extended from September 2021 to September 2023, the findings should be interpreted in the context of the later phases and aftermath of the COVID-19 crisis rather than in the acute initial outbreak period. Future longitudinal research is warranted to clarify the temporal relationships between psychological distress, resilience, and quality of life, and to evaluate the effectiveness of targeted interventions in vulnerable populations.

## 5. Conclusions

This study identifies significant correlations between fear of COVID-19, psychological distress, and quality of life among patients with chronic conditions, across the later phases and aftermath of the COVID-19 crisis. The results suggest that fear of COVID-19, psychological distress, resilience, and quality of life are interrelated in a complex manner. While higher fear levels are associated with diminished well-being, the potential for a bidirectional relationship between fear and quality of life remains a critical consideration.

The findings underscore the importance of psychological resilience and social support in this population. However, to move beyond preliminary associations and definitively establish the causal mechanisms and directionality of these links, future longitudinal research is essential. These insights are vital for developing integrated healthcare strategies that address both the physical and psychological needs of individuals with chronic conditions during public health crises and their aftermath.

## 6. Limitations

The study has several limitations that should be considered when interpreting the results. First, the use of a self-recruited convenience sample of hospital patients may introduce selection bias, as participants who volunteered may differ in health literacy or motivation compared to those who did not. Second, while the General Hospital of Argolida serves a catchment area of tens of thousands, our sample size of *N* = 293 represents a relatively small proportion of the total patient population. This may limit the generalizability (external validity) of the findings to the broader regional population. Although a post hoc power analysis suggested that the sample was adequate to detect medium effect sizes within the study sample, this does not eliminate the limitations associated with convenience sampling or representativeness. Future research should ideally employ random sampling methods and include multiple clinical sites to ensure a more representative demographic profile. A potential limitation of this study is the inclusion of specific pandemic-related questions (e.g., the expected duration of the crisis) during a two-year window that spanned both the later phases of the pandemic and the transition to the post-pandemic period. As the WHO officially ended the global public health emergency in May 2023, the final months of our data collection occurred in a different social context. For patients with chronic conditions, however, the transition out of the pandemic period may have been experienced differently due to ongoing health vulnerabilities, and this should be considered when interpreting responses related to the perceived resolution of the COVID-19 crisis. Finally, the cross-sectional design of this research precludes any conclusions regarding causality. Although regression models were used to explore associations with quality of life, the direction of the identified associations cannot be determined, and bidirectional relationships are possible. Future longitudinal studies are required to establish the temporal sequence and causal direction between psychological stressors and health-related quality of life in individuals with chronic conditions.

## Figures and Tables

**Table 1:** Sociodemographic, clinical, lifestyle, and COVID-19-related characteristics of the study population (*N* = 293).

Category	Characteristic	n (%)
Gender	Male	130 (44.4)
	Female	163 (55.6)
Age Group	18–40 years old	113 (38.7)
	41–59 years old	108 (37.0)
	≥60 years	71 (24.3)
Family Status	Married or living with partner	165 (56.5)
	Has at least one child	166 (58.9)
	Household with ≥3 members	153 (52.2)
Education	Secondary education	101 (34.5)
	Primary or lower secondary	89 (30.4)
Employment Status	Unemployed	76 (26.4)
	Retired	64 (22.2)
	Public sector employee	49 (17.0)
	Private sector employee	41 (14.2)
Work during COVID-19 *	Continued on-site	75 (52.8)
	Remote work	24 (16.9)
	Work suspension	23 (16.2)
Residence	Urban settings (town/city)	196 (67.8)
Health Status	Chronic physical condition	182 (62.5)
	Long-standing mental health disorder	84 (29.0)
Healthcare Adherence	Adherence to medical treatment	236 (87.1)
	Routine follow-up attendance	224 (82.4)
	Monitoring in private facilities	136 (52.5)
Lifestyle Factors	Sedentary lifestyle	152 (53.0)
	Current smoker	117 (40.1)
	Weekly alcohol (1–2 beverages)	81 (28.0)
	Exercise > 3 h/week	98 (33.8)
Technology Use	Mobile phone use > 3 h/day	120 (41.2)
	Computer use > 3 h/day	97 (33.4)
Primary Support Source	Spouse	114 (38.9)
	Friends	74 (25.3)
	Children	59 (20.1)
COVID-19 variables	History of COVID-19 infection	20 (6.9)
	COVID-19 vaccination	149 (51.4)

* Answered only by participants who were employed.

**Table 2:** Descriptive statistics and internal consistency coefficients for MVQOLI, Fear of COVID-19, DASS-21, and resilience scales.

Scale/Domain	Minimum	Maximum	Mean (SD)	Median (IQR)	Cronbach’s α
Symptom	−24.0	30.0	7.4 (8.4)	8 (2–12)	0.71
Function	−30.0	30.0	3.3 (10.0)	4 (−4–9)	0.63
Interpersonal	−30.0	30.0	9.2 (13.1)	12 (0–16)	0.64
Well-being	−30.0	30.0	−2.4 (11.8)	−4 (−10–6)	0.67
Transcendence	−30.0	30.0	10.1 (11.9)	12 (2–20)	0.72
Total MVQOLI score	10.0	24.9	17.8 (2.8)	18 (16–19.4)	0.77
Fear of COVID-19	7.0	35.0	16.8 (6.0)	16 (13–21)	0.88
Depression	0.0	21.0	4.8 (5.5)	3 (0–8)	0.92
Anxiety	0.0	21.0	4.2 (4.9)	3 (0–7)	0.87
Stress	0.0	21.0	6.1 (5.2)	5 (2–9.5)	0.89
Total DASS-21	0.0	63.0	15.0 (14.8)	10 (3–22)	0.92
Resilience scale	11.0	100.0	68.5 (16.8)	68.8 (60–80)	0.91

Note: Each MVQOLI domain score ranges from −30 to +30, while the total MVQOLI score ranges from 0 to 30; higher scores indicate better overall quality of life.

**Table 3:** Spearman correlation coefficients between MVQOLI domains, fear of COVID-19, psychological distress, and resilience.

MVQOLI Domains	Fear of COVID-19	Resilience Scale	Depression	Anxiety	Stress	Total DASS-21 Score
Symptom	0.06	0.07	−0.07	−0.12	−0.08	−0.10
Function	−0.25 ***	−0.06	−0.27 ***	−0.12	−0.12	−0.16 *
Interpersonal	−0.13 *	0.27 ***	−0.28 ***	−0.19 **	−0.13 *	−0.18 **
Well-being	−0.24 ***	0.01	0.04	0.05	0.05	0.05
Transcendence	−0.21 **	0.28 ***	−0.22 ***	−0.19 **	−0.10	−0.17 **
Total MVQOLI score	−0.14 *	0.28 ***	−0.23 ***	−0.17 **	−0.08	−0.14 *

* *p* < 0.05; ** *p* < 0.01; *** *p* < 0.001.

**Table 4:** Multiple linear regression analyses for MVQOLI domains and total MVQOLI score.

	β +	SE ++	P
Symptom (R^2^ = 0.11, *N* = 272)			
Area of residence: Village/island/other vs. small/large city	0.038	0.018	0.044
Function (R^2^ = 0.13, *N* = 237)			
Fear of COVID-19	−0.005	0.002	0.008
Depression	−0.003	0.001	0.017
Interpersonal (R^2^ = 0.11, *N* = 219)			
Depression	−0.011	0.003	<0.001
Resilience scale	0.002	0.001	0.046
Well-being (R^2^ = 0.10, *N* = 259)			
Fear of COVID-19	−0.006	0.003	0.046
Transcendence (R^2^ = 0.13, *N* = 255)			
Depression	−0.009	0.002	<0.001
Chronic physical illness (Yes vs. No)	−0.053	−0.024	0.029
Resilience scale	0.002	0.001	0.001
Total MVQOLI score (R^2^ = 0.12, *N* = 229)			
Depression	−0.010	0.002	<0.001
Resilience scale	0.003	−0.001	0.002

Note: + regression coefficient ++ Standard Error. Note: Logarithmic transformation of the dependent variable was used for this analysis.

## Data Availability

The data presented in this study are available from the corresponding authors upon reasonable request.

## Abbreviations

The following abbreviations are used in this manuscript:

CD-RISC-25	Connor–Davidson Resilience Scale (25-item version)
COVID-19	Coronavirus Disease 2019
DASS-21	Depression Anxiety Stress Scale (21-item version)
FCV-19S	Fear of COVID-19 Scale
MVQOLI	Missoula–Vitas Quality of Life Index
QoL	Quality of Life
